# Birth preparedness and complication readiness in Robe Woreda, Arsi Zone, Oromia Region, Central Ethiopia: a cross-sectional study

**DOI:** 10.1186/1742-4755-11-55

**Published:** 2014-07-20

**Authors:** Muhammedawel Kaso, Mesfin Addisse

**Affiliations:** 1 College of Medicine and Health Sciences, Madawalabu University, Bale-Goba, Ethiopia; 2 School of Public Health, Addis Ababa University, Addis Ababa, Ethiopia

**Keywords:** Birth preparedness, Complication readiness, Place of delivery, Skilled attendant, Arsi zone, Antenatal care

## Abstract

**Background:**

Globally, an estimated 287 000 maternal deaths occurred in 2010 annually as a result of complications of pregnancy and childbirth. Sub-Saharan Africa and Southern Asia were accounted for 85% of the global burden (245 000 maternal deaths) including Ethiopia. Obstetric related complications cannot be reliably predicted. Hence, insignificant decline of maternal mortality ratio might be due to the non use of birth preparedness and complication readiness strategies. Therefore, this paper aimed to assess knowledge and practices towards birth preparedness and complication readiness and associated factors among women of reproductive age group (15–49) in Robe Woreda, Arsi Zone, Oromia Region, Ethiopia.

**Method:**

Community-based cross-sectional study supplemented by qualitative design was conducted in January, 2012. A total of 575 women from 5 kebeles were selected after proportionally allocated to population size and interviewed using structured and semi-structured, pre-tested questionnaires. Univariate and bivariate analysis was performed. Multiple logistic regression analysis was also done to control for possible confounding variables.

**Results:**

Taking into account place of delivery identification, means of transportation, skilled attendant identification and saving money, about 16.5% of the respondents were prepared for birth and its complications. Preparation for birth and its complication was higher among educated mothers (AOR = 6.23, 95% CI = 1.5, 25.87). Monthly income of >716 Ethiopian birr (AOR = 1.94, 95% CI = 1.01, 3.87), ANC visit (AOR = 5.68, 95% CI = 1.27, 25.4), knowledge of obstetric complications (AOR = 2.94, 95% CI = 1.61, 5.37) and those who had given birth at health facility before their last delivery (AOR = 3.9, 95% CI = 2.04, 7.46) were also significantly associated with birth preparedness and complication readiness.

**Conclusion:**

The study identified very low magnitude of birth preparedness and complication readiness in the study area and poor knowledge and practices of preparation for birth and its complication. Community education about preparation for birth and its complication and empowerment of women through expansion of educational opportunities are important steps in improving birth preparedness. In all health facilities during antenatal care emphasis should given to preparation for birth and its complication and provide information and education to all pregnant women.

## Background

Maternal mortality is a substantial problem in developing countries [1]. Decreasing maternal mortality has got recognition at global level as evidenced by the inclusion of reducing maternal mortality in the Millennium Development Goals [2].

Birth preparedness is a comprehensive strategy to improve the use of skilled providers at birth and the key intervention to decrease maternal mortality. Birth preparedness and complication readiness (BP/CR) is the process of planning for normal birth and anticipating actions needed in case of emergency. It encourages women, households, and communities to make arrangements such as identifying or establishing available transport, setting aside money to pay for service fees and transport, and identifying blood donor in order to facilitate swift decision-making and reduce delays in reaching care once a problem arises. Responsibilities for BP/CR must be shared among all safe motherhood stakeholders, since coordinated effort is needed to reduce the delays that contribute to maternal and newborn deaths [1].

Too often, however, their access to care is impeded by delays; delays in deciding to seek care, delays in reaching care and delays in receiving care. These delays have many causes; including logistic and financial concerns, unsupportive policies and gaps in services, as well as inadequate community and family awareness and knowledge about obstetric complication issues [2].

Maternal and neonatal mortality and morbidity rates in Ethiopia are among the highest in the world and stem from a range of socio-economic, political and demographic factors. Many of these deaths are preventable and include complications such as hemorrhage, infection, obstructed labour, abortion and eclampsia in mothers and asphyxia, infection and prematurity or low birth weight among the new born [3]. Pregnancy and childbirth and their complications are the leading causes of death, disease and disability among women of reproductive age in developing countries more than any other single health problem [1].

Globally, an estimated 287 000 maternal deaths occurred in 2010 annually as a result of complications of pregnancy and childbirth. Sub-Saharan Africa and Southern Asia were accounted for 85% of the global burden (245 000 maternal deaths) including Ethiopia [2,3]. Pregnancy related complications cannot be reliably predicted and it is necessary to design strategies to overcome those problems when they arise [1,4,5].

Worldwide more than 70% of all maternal deaths are due to five major complications: hemorrhage, infection, unsafe abortion, hypertensive disorders of pregnancy and obstructed labor. An estimated 40% of pregnant women (50 million per year) were experienced pregnancy-related health problems during or after pregnancy and childbirth with 15% suffering serious or long term complications. As a consequence, 300 million women suffer from pregnancy-related health problems and disabilities; including anemia, uterine prolapse, fistula, pelvic inflammatory disease (PID), and infertility [6-9].

In Ethiopia direct obstetric complication accounts for 85% of the deaths. It includes abortion 32%, obstructed labor 22%, sepsis 12%, hemorrhage 10% and hypertension 9%, primarily due to frequency of adolescent pregnancy combined with neglected prolonged labor [2,5,7]. Most of these deaths are preventable when there is access to adequate reproductive health services, equipment, supplies and skilled healthcare workers [3,4].

The MMR in developing regions (240) was 15 times higher than in developed regions. Sub-Saharan Africa had the highest MMR at 500 maternal deaths per 100, 000 live births, while Eastern Asia had the lowest among MDG developing regions, at 37 maternal deaths per 100, 000 live births. In sub-Saharan Africa, a woman’s maternal mortality risk is 1 in 30, compared to 1 in 5,600 in developed regions [3-5].

In Millennium Development Goal 5, countries are committed to reducing the maternal mortality ratio by three quarters between 1990 and 2015. Following this commitment, Ethiopia is expected to reduce maternal mortality in 2015 to 267 maternal deaths per 100,000 live births [4,5].

But according to 2011Ethiopian Demographic and Health Survey report, the maternal mortality ratio was 676 maternal deaths per 100,000 live births for the seven year period preceding the survey. This ratio is not significantly different from those reported in the 2005 EDHS and 2000 EDHS.

An estimated maternal mortality ratio is almost the same in the 2011 EDHS (676) as it was in the 2005 EDHS (673). The confidence interval surrounding the maternal mortality ratio of 676 deaths per 100,000 live births is 541–810, while the confidence interval for the 2005 ratio of 673 deaths per 100,000 live births is 548–799 deaths. Because of confidence intervals between the two estimates overlap, there is no evidence to suggest that maternal mortality ratio changed in the six years between surveys. A similar conclusion can be drawn comparing the maternal mortality ratios measured in the 2011 EDHS with those in the 2000 EDHS which was 871 deaths per 100,000 live births.

Thus, there is no evidence to suggest that maternal mortality ratio was decreased in Ethiopia between 2000 and 2011. This insignificant decline of maternal mortality ratio might be due to the non use of birth preparedness and complication readiness strategies associated with income, educational status, knowledge of obstetric complications, perception and ANC attendance [10,11]. Birth preparedness and complication readiness is less common in many developing countries including Ethiopia. For example, 22.0% of women in northern Ethiopia, 27.5% in Northern Nigeria and 17% in Southern Ethiopia were prepared for birth and its complication [10-12].

Meta-analyses showed that exposure to BP/CR interventions was associated with a statistically significant reduction of 18% in neonatal mortality risk (twelve studies, RR = 0.82; 95% CI: 0.74, 0.91) and a non-significant reduction of 28% in maternal mortality risk (seven studies, RR = 0.72; 95% CI: 0.46, 1.13) [13].

Despite the fact that BP/CR is essential for further improvement of maternal and child health and prevention of maternal deaths, little is known about the status of birth preparedness and complication readiness in rural Ethiopia in general and in Oromia Region in particular. Therefore, this paper was aimed to assess knowledge and practices with respect to birth preparedness and complication readiness and factors associated in rural community among women of reproductive age in Robe Woreda, Arsi Zone, Oromia region, Ethiopia. The results of the study will provide valuable information for design of possible programs and interventions to improve maternal and neonatal health. And also serve as baseline information for further study.

## Methods

Community-based cross-sectional study supplemented by qualitative design was conducted in January, 2012 among women who gave birth in the last 12 months preceding the survey irrespective of birth outcome in Robe Woreda, Oromia Region, central Ethiopia. Robe Woreda is located 223 km east of Addis Ababa. The Woreda is divided in to 4 admistrative towns (Robe; capital town of Woreda, Habe, Sadika and Endato) and 28 rural kebeles. The total population in the Woreda is estimated to be 184,367 with an area of 127.5 square kilometers. It has one district government hospital, four health centers, 28 health posts and 11 private clinics.

The sample size was calculated using the formula for single population proportion. Accordingly, a total of 581 women was determined as a sample size based on assumption of confidence level of 95%, margin of error 5%, 22% proportion of birth preparedness and complication readiness among women in Adigrat town, Tigray region, northern Ethiopia (10), considering a design effect of 2 and a non-response rate of 10%.

For the qualitative methods, a total of five traditional birth attendants (TBAs) for in depth interview and four FGDs, each group contains 6 women were enrolled and interviewed using open ended questions related to the objective of the study.

To identify the study units, for the quantitative data, the study employed cluster sampling technique. Based on the catchment areas of each 4 health centers in the Woreda (since these health centers are assumed to be distributed evenly in the Woreda), all rural Kebeles in the Woreda were grouped in to four groups (clusters) and then, one cluster was selected by simple random sampling techniques.

The final sample size was proportionally allocated to each kebele after the population size in the kebele was identified. After selecting a random starting point, individuals were selected in the kebele till the desired sample size of individuals was reached in each kebele. For households’ which had more than one eligible woman, interview was done by selecting a woman using lottery method. In the event of a household with no eligible woman, the immediate next household with eligible woman was interviewed. Women of permanent resident of the study area, volunteer to participate and respond to the questionnaire were included. Women who were mentally disabled and severely ill were excluded.

For the qualitative design, purposive sampling technique was employed for an in depth interview of one TBA from each kebele. For FGDs women in the age group (15–49) who were locally respected was selected purposively from two kebeles. Participants interviewed in the quantitative survey were excluded from the FGD. Age of the participants was considered for grouping of the participants in the FGD. Two FGDs (one group 15–30 years and the other group >30 years) were taken from each kebele after the two kebeles were selected by simple random sampling techniques among kebeles in the selected cluster and discussion were conducted based on information saturation.

A woman was considered as prepared for birth and its complication if she reported that she identified place of delivery, saved money, identify skilled provider at birth and identified a means of transport to place of childbirth or for the time of obstetric emergencies ahead of childbirth. These four variables were transformed on SPSS into a single variable, that is “birth preparedness and complication readiness” which was identified whether the woman prepared for birth and its complication or not. Then those mothers who followed at least three of the four BP/CR components were considered as “prepared for birth and its complication”. The remaining women were considered as “not prepared for birth and its complication”.

A woman was considered knowledgeable on key danger signs of pregnancy, if she can mention at least two of the three key danger signs for pregnancy (vaginal bleeding, swollen hands/face and blurred vision) spontaneously. A woman was considered knowledgeable on key danger signs of labour/childbirth, if she can mention at least three of the key four danger signs for labour/childbirth (severe vaginal bleeding, prolonged labour (>12huors), convulsion and retained placenta) spontaneously. A woman was considered knowledgeable on key danger signs of postpartum, if she can mention at least two of the three key danger signs for postpartum (severe vaginal bleeding, foul-smelling vaginal discharge and high fever) spontaneously.

The structured questionnaires mainly adapted from monitoring birth preparedness and complication readiness, tools and indicators for maternal and newborn health (1) was used in English. The principal investigator translated the English version to the local language (Afan Oromo). Then another individual who had very good knowledge of both English and Afan Oromo language then translated the Afan Oromo version back to English to check for its original meaning.

The questionnaires were pre-tested on kebele’s that were similar characteristics with the study population other than the sampled cluster in the study area. One kebele was selected randomly for this purpose. A total of 29 respondents (5% of sample size) were interviewed. Findings were discussed among data collectors and supervisors in order to ensure better understanding to the data collection process. Based on the pretest, questions were revised, edited, and those found to be unclear or confusing were modified. Finally, structured closed ended Afan Oromo version questionnaire was used for data collection.

Five diploma nurses who were fluent in Afan Oromo language collected the data. Two supervisors with BSc nurse background were supervised the data collectors. They have been trained for two days on the study instrument and data collection procedures. Data were collected through face-to-face interview with the study subjects. Codes were given to the questionnaires and household during data collection. Filled questionnaires were checked daily for completeness, legibility and consistency. Incomplete and unclear questionnaires were returned back to the interviewers to get it complete for the next day using the codes. Consequently, any problem encountered was discussed among the survey team and solved immediately.

The survey was conducted after approval by the Institutional Review board of School of Public Health, Addis Ababa University. Official letter was obtained from the School of Public Health of Addis Ababa University to respective Woreda. Permission to carry out the study was obtained from the Woreda Health Bureau. Individual informed verbal consent was obtained from each respondent after explaining the objective and procedures of the study. The information was kept confidential. At the end of the interview, health information regarding risks of home delivery, possible complications of pregnancy and how to respond to them was provided as deemed necessary.

FGD moderators (Principal Investigator and one note taker) and in depth interview interviewer (Principal Investigator) explained the purpose of the study and obtained voluntary informed consent prior to conducting the discussion. Note taking and tape recorder were used for in depth interviews and FGDs.

Data first were checked manually for completeness then coded, prepared template and entered into Epi-Info version 3.5.1statistical software and cleaned thoroughly before transferred to SPSS version 16 for further analysis. The data was further cleaned by visualizing, double entry on 10% of questionnaires, calculating frequencies and sorting outliers. Corrections were made according to the original data and cleaned data were exported from Epi Info version 3.5.1 to SPSS version 16.0 for analysis.

Univariate analysis was done using frequency, percentage and presented in the form of texts and tables. Logistic regression analysis was done to identify factors associated with BP/CR. Binary analysis was used to determine the association between different factors and the outcome variable. Multiple logistic regression analysis was done to control for possible confounding variables. Those variables which were showed significant association on bivariate analysis (P-value < 0.05) were adjusted to each other to identify independent associated variables. P-value and 95% confidence interval (CI) for OR were used in judging the significance of the associations. P-value less than 0.05 were taken as a cut of point to declare significant association. The qualitative data was transcribed into English text by the principal investigator by replaying the recorded interview. Different ideas in the text were merged in their thematic areas and a thematic framework analysis was employed manually. Then, the result was presented in narration by triangulating with quantitative findings.

## Results

### Socio-demographic characteristics of study population

A total of 575 women out of 581, who gave birth in the past twelve months prior to this study were interviewed making a response rate of 99%. One hundred thirty five (23.5%) were less than 20 years while elderly gravida (35 years and above) were 21 (3.7%). The mean age of respondents was 25 ± 4.6 years, with range of 14–38 years (Table 1).

**Table 1:** Socio-demographic characteristics of respondents among women aged 15–49 years, Robe Woreda, January, 2012

**Variables**	**Frequency**	**Percent**
**Age of respondents**		
<20	135	23.5
20–34	419	72.9
35–49	21	3.7
**Marital status**		
Married	545	94.8
Separated	16	2.8
Widowed	7	1.2
Single	4	0.7
Divorced	3	0.5
**Religion**		
Muslim	446	77.6
Orthodox	129	22.4
**Ethnicity**		
Oromo	545	94.8
Amhara	30	5.2
**Educational status**		
Illiterate	398	69.2
Read and write	34	5.9
Primary education	132	23
Secondary education & above	11	1.9
**Occupational status**		
Housewife	562	97.7
Farmer	4	0.7
Private employee	1	0.2
Merchant	8	1.4
**Monthly income**		
< 131 Birr	143	24.9
131–475 Birr	146	25.4
476–716 Birr	143	24.9
>716	143	24.9
**Family size**		
1–3	83	14.4
4–6	285	49.6
> = 7	207	36

### Obstetric history and ANC experience of the respondents

The majority (86.4%) of the respondents have attended antenatal care (ANC) at least once in their last pregnancy period. Out of the total mothers who have attended ANC, 49% of the respondents started their follow up while the pregnancy was between 4 and 6 months and 31% of the respondents had their first ANC visit in the first three months of pregnancy. One hundred thirty nine (28%) of the respondents had 4 or more visits. The reasons given by the mother’s who follow-up ANC were; for their health in general 226(39.8%), to know the condition of pregnancy 119 (20.7%), for vaccination only 106 (18.4%), for vaccination and checkup 62 (10.8%) and for nutrition advice 13 (2.3%). Some, 46 (8%) of the mothers didn’t know the purpose of ANC follow-up while they follow-up ANC.

For 95 (16.5%) of the mothers, the last pregnancy was their first and 188 (32.7%) of them had 5 and above pregnancies. Among the respondents 88 (16.8%) had one child, 276 (52.7%) had 2–4 children and 160 (30.5%) had > = 5 children with mean parity of 3.6 ± 2.1.

Majority of (89.6%) the women were became pregnant at the age of their <20 years and 60 (10.4%) were became pregnant between the age of 21–29 years. The mean age of the women was 17.27 ± 2.95 years. Fifty one (8.9%) of the respondents had history of abortion whereas 1.6% had history of still birth.

Five hundred twenty two (90.8%) women were gave birth at home whereas 9.2% gave birth in the health institutions. Among the respondents sixty four (11.1%) women gave birth at health facility before their last delivery while 7 (1.2%) of the women gave birth by Cesarean section.

The women who gave birth in the home were give the reasons which includes; short and smooth labor 514 (98.5%), normal previous home delivery 185 (35.4%), too much cost of health facility 7 (1.3%), presence of TBAs 6 (1.1%), perceived poor quality service of health facility 3 (0.6%), informed that their pregnancy was normal 2 (0.4%), health facility too far 2 (0.4%) and other reasons 4 (0.8%).

Reasons given for institutional delivery among those who gave birth at health facility were, difficult labor 32 (60.4%), need better service in health facility 14 (26.4%), informed to deliver in health facilities 13 (24.5%), Poor out comes from previous home delivery11 (20.8%), previous better outcomes from Institutional delivery 4 (7.5%) and health facility close to where they live 2 (3.8%).

### Knowledge of respondents about danger signs during pregnancy

Relatively low proportion, 217 (37.7%), 58 (10.1%) and 36 (2.2%) of the respondents spontaneously mentioned blurred vision, vaginal bleeding and swollen hands/face as danger signs during pregnancy, respectively. Two hundred thirty one (40.2%) of the respondents were spontaneously mentioned at least one key danger sign, 75 (13%) mentioned at least two key danger signs and 5 (0.9%) mentioned all three key danger signs.

### Knowledge on danger signs during labor/childbirth

Three hundred sixty (55%), 244 (42.4%), 153 (26.6%) and 66 (11.5%) of the respondents spontaneously mentioned prolonged labor, retained placenta, severe vaginal bleeding and convulsions as danger signs during labor and childbirth respectively. Four hundred fifty six (79.3%) respondents spontaneously mentioned at least one key danger sign, 244 (42.4%) mentioned at least two key danger signs while 75 (13%) cited at least three key danger signs. Only four (0.7%) respondents named all four key danger signs.

### Knowledge on danger signs during post partum period

One hundred ninety six (34.1%), 59 (10.3%) and 49 (8.5%) of the respondents spontaneously mentioned severe vaginal bleeding, foul smelling vaginal discharge and high fever as danger signs during post partum period, respectively. Two hundred forty eight (43.2%) of the study participants spontaneously mentioned at least one key danger sign, 55 (9.6%) mentioned at least two key danger signs and one (0.2%) mentioned all three key danger signs.

Some of the TBAs in in-depth interviews and mothers in focus group discussion have mentioned some of the danger signs during pregnancy, delivery and post-partum period.

The majority of TBA’s interviewed explained the issue using the following expression or one similar to it:

“I said that when the mothers developed vaginal bleeding, prolonged labor and retained placenta, she would be in the serious problems or in danger condition ………especially if there was a retained placenta it was a severe one, because the placenta goes to her head and kill immediately.” (56 years old mother)

Mothers addressed the issue in this way:

“I said when mothers developed dizziness, vaginal bleeding, vomiting and retained placenta; she would be in a dangerous health problems.” (30 years old mother)

### Knowledge of respondents about preparation for birth and its complication

Five hundred sixty one (97.6%), 401 (69.7%), 394 (68.5%), 292 (50.8%) and 57 (9.9%) of the respondents were spontaneously mentioned as it is useful for birth and its complication when saving money, identified means of transportation, identified skilled provider, identified place of delivery and blood donor respectively.

Majority (63.8%) of the respondents were agreed that there were any danger signs that can occur during pregnancy, labour and post-partum while 208 (36.2%) of the respondents did not report any danger signs that can occur during pregnancy, delivery and post-partum period. Among study participants, 13% of respondents were knowledgeable on danger signs during pregnancy while 13% of respondents were knowledgeable on danger signs during delivery. Only 9.6% of respondents had knowledge on danger signs of post-partum period (Table 2).

**Table 2:** Knowledge of respondents about preparation for birth and its complication among women aged 15–49 years, Robe Woreda, January, 2012

**Variables**	**Frequency**	**Percent**
**Identify place of delivery**		
Yes	292	50.8
No	283	49.2
**Saving money**		
Yes	561	97.6
No	14	2.4
**Prepare essential items for child birth**		
Yes	574	99.8
No	1	0.2
**Identify skilled provider**		
Yes	394	68.5
No	181	31.5
**Awareness on the danger signs of:-**		
Pregnancy	75	13
Delivery	75	13
Post-partum	55	9.6
**Identify a mode of transportation**		
Yes	401	69.7
No	174	30.3
**Arranging blood donors**		
Yes	57	9.9
No	518	90.1
**Obstetric danger signs during pregnancy, labour and post-partum**		
Yes	367	63.8
No	208	36.2

Blurred vision was the most known danger sign in pregnancy spontaneously mentioned by 37.7% of the respondents, followed by hemorrhage which spontaneously mentioned by 10.1% of the respondents. Severe vaginal bleeding and swelling of hands and face were known by very few respondents. Severe vaginal bleeding was reported by 26.6% and 34.1% of the respondents during delivery and post-partum period respectively.

Maternal knowledge on severe vaginal bleeding during pregnancy, delivery and post-partum period were transformed on SPSS into a single variable. Most (45.6%) of the respondents didn’t mention severe vaginal bleeding spontaneously as a danger sign during pregnancy, delivery and post-partum period. Among study participants, almost half (54.4%) of respondents mentioned severe vaginal bleeding at least in one of the three periods while 13.6% mentioned spontaneously in two of the three periods. Only 2.8% of respondents mentioned severe vaginal bleeding spontaneously as a danger sign during pregnancy, delivery and post-partum period.

### Practices of respondents regarding preparation for birth and its complication

Almost all (99.1%) of the respondents reported that they made some arrangement for birth during their last delivery. Some of preparations reported by the mothers were food; like grain for porridge, butter, honey and sheep and a new blade for cord cutting, cloths for newborn, cleaning the room where delivery would be conducted and identified helper during delivery. Among the total respondents, there were women who reported that they made critical preparations which are very important components of birth preparedness and complication readiness such as arranging transportation, identifying an institution for delivery, identified skilled attendant and saving money.

Four hundred thirty nine (76.3%) of the respondents reported spontaneously that they saved money, 262 (45.6%) identified place of delivery, 164 (28.5%) identified a mode of transportation and 17 (3%) identified skilled provider. But, among mother’s who identified place of delivery (45.6%), majority of the mothers (40.7%) planned to give birth at home while only (4.9%) of the mothers planned to give birth at the nearest health facility. Considering those important components, birth preparedness and complication readiness among the age group 15–49 years with birth in the one year prior to the survey was 16.5%.

Of the 575 mothers, 78 (13.6%) experienced delivery- related complication (s) cited earlier; 54 (69.2%) of the 78 mothers were delivered by a skilled birth attendant, whereas 24 (30.8%) did not go to health facilities to gain the delivery services.

In focus group discussions and in-depth interviews of mothers and TBA’s that were mentioned problems related to referral system, majority of the mothers and TBA’s interviewed explained the condition. Now a day if there were problems and told to them to go to health facilities, the mothers will go to health facility without delays. Even if she had no any money for transport and other payment, she would be supported by relatives and communities and carried by people to health facility.

Majority of TBA’s and mothers interviewed were explained using the following expression:

*“If problems occurred and told to them to go to health facility they directly go to health facility. Mothers who have no money for transport and other cost are supported and carried by people. We know that there is no need of payment for normal delivery. If may be operation was needed, there were payment that was contributed and paid by the communities.” (45 years old mother*)

In qualitative part of in-depth interviews and focus group discussions there were mothers and TBA’s interviewed.

Majority of TBA’s and mothers mentioned the most common maternal problems in the area were blurred vision, retained placenta and prolonged labor. Some TBA’s and mothers also explained distance of health facility is the major challenge for mothers in the area.

One TBA from one kebele mentioned the most common maternal health problems in the area as follows:

“Problems that mothers faced were blurred vision and prolonged labor. Also too far health facility is the major challenge for mothers in our area.” (60 years old mother)

In focus group discussions and in-depth interviews mothers and TBA’s were mentioned traditional practices done in the area by the mothers or the community especially during pregnancy, childbirth and immediately after delivery assuming it will benefit the mother’s health.

Among TBA’s those interviewed one expressed the issue as follows:

“In our culture we gave for mothers to drink old/fermented butter to clean out her uterus from clotting blood…………..also immediately we gave for newborn a fresh butter after birth, because till the mother’s breast got enough milk it is used for new born as a food………………..still I believe all those activities are useful for newborn and mothers as we have seen even from experiences.” (40 years old mother)

Mother’s especially younger mothers were more explained traditional practices positively. They mentioned those traditional practices like giving for newborn anything before six months, cutting uvula and other harmful practices were forbidden and affects mothers and newborn health.

Among mothers of 15–30 years old of focus group discussion one mother expressed the issue as follows:

“In the past time our mothers gave fresh butter for newborn, cutting uvula and even circumcised……but, now a day HEWs tells us not to give anything for newborn before six months.” (26 years old)

Finally, the mothers and TBAs recommended that government should give the services for the mothers; especially during delivery the service should be free for the mothers. Furthermore, ambulance should prepare for the mothers for emergency situation.

### Factors influencing birth preparedness and complication readiness

#### Socio-demographic factors and past obstetric experiences

In bivariate analysis maternal education was significantly associated with birth preparedness and complication readiness. Mothers whose educational status was secondary high school and above were about 10 times more likely to prepare for birth and it’s complication than women with other levels of education (COR = 10.06, 95% CI: 2.85, 35.42). This study revealed that women who had incomes of > = 717 Ethiopian birr were about two times more likely to prepare for birth and its complication than women having incomes of less than 716 birr (COR = 2.1, 95% CI: 1.14, 3.83).

Knowledge of danger sign of obstetric complications was also significantly associated with birth preparedness and complication readiness. Mothers who know the presence of obstetric complications were three times more likely to prepare for birth and its complications than mothers who didn’t know the presence of complications (COR = 3.03, 95% CI: 1.74, 5.29).

Birth order of four or more and being grand multipara found to be significantly associated with birth preparedness and complication readiness. As birth order increases birth preparedness and complication readiness decreases (COR = 0.455, 95% CI: 0.25, 0.82). As gravidity and parity increases more than five pregnancy/child birth, birth preparedness and complication readiness decreases (COR = 0.42, 95% CI: 0.22, 0.79 and COR = 0.41, 95% CI: 0.22, 0.77) respectively.

Obviously, prenatal visit was found to be factors associated with birth preparedness and complication readiness. Women who had history of antenatal visit were more likely to prepare for birth and its complication (COR = 8.75, 95% CI: 2.11, 36.26). Frequency of antenatal care follow-up also found to be strong predictor of birth preparedness and complication readiness (COR = 11.51, 95% CI: 2.39, 55.47). Mothers who had given birth at health facility before their last delivery were more likely to prepare for birth and its complication (COR = 3.72, 95% CI: 2.11, 6.54).

Moreover, mothers who had past history of still birth were more likely to prepare for birth and its complication (COR = 4.18, 95% CI: 1.1, 15.85).

#### Multivariate analysis of socio- demographic and obstetric influencing factors on birth preparedness and complication readiness adjusted for possible confounding variables

By Applying multiple logistic regression on socio demographic variables; women’s education and monthly income and obstetric factors; gravida, parity, ANC visit, knowledge of the danger signs of obstetric complications, presence of history of still birth, history of delivery at health facility before last delivery, and birth order were adjusted. Only their educational status and their monthly income were significantly associated with birth preparedness and complication readiness among socio-demographic variables. If women have secondary education and above six times and monthly income above 716 Ethiopian birr two times were more likely to prepare for birth and its complication (AOR = 6.23 and 95% CI = 1.5-25.87) and (AOR = 1.97 and 95% CI = 1.01-3.87) respectively. Also mothers who know the presence of obstetric complications were three times more likely to prepare for birth and its complications than mothers didn’t know the presence of complications (AOR = 2.94, 95% CI: 1.61, 5.37) (Table 3).

**Table 3:** Socio-demographic and obstetric factors influencing birth preparedness and complication readiness adjusted for confounding variables, in Robe Woreda, January, 2012

**Variable**	**Birth preparedness**		**COR (95% CI)**	**AOR (95% CI)**
	**Yes**	**No**		
**Women’s education**				
Illiterate	59 (62.1%)	339 (70.6%)	1.00	1.00
Read and write	1 (1.1%)	33 (6.9%)	0.17 (0.02, 1.3)	0.15 (0.02, 1.2)
Primary education	28 (29.5%)	104 (21.7%)	1.55 (0.94, 2.55)	1.36 (0.76, 2.43)
Secondary education & above	7 (7.4%)	4 (0.8%)	10.1 (2.85, 35.42)*	6.23 (1.45, 25.87)**
**Monthly income**				
< 131 Birr	20 (21.1%)	123 (25.7%)	1.00	1.00
131–475 Birr	18 (18.9%)	128 (26.7%)	0.87 (0.44, 1.7)	0.98 (0.46, 2.06)
476–716 Birr	21 (22.1%)	122 (25.5%)	1.06 (0.55, 2.05)	1.11 (0.54, 2.3)
>716	36 (37.9%)	106 (22.1%)	2.1 (1.14, 3.83)*	1.97 (1.01, 3.9)**
**Gravida (total no. of pregnancy)**				
1	23	72	1.00	1.00
2–4	50	242	0.65 (0.37, 1.13)	5.49 (0.32, 94.87)
> = 5	22	166	0.42 (0.22, 0.79)*	0.44 (0.006, 31.66)
**Parity (total no. of birth)**				
1	26	79	1.00	1.00
2–4	48	245	0.59 (0.35, 1.02)	0.97 (0.17, 5.53)
> = 5	21	156	0.41 (0.22, 0.77)*	5.39 (0.16, 184.59)
**ANC visit**				
Yes	93	404	8.75 (2.11, 36.26)*	5.68 (1.27, 25.4)**
No	2	76	1.00	1.00
**Obstetric danger signs during pregnancy, labour and post-partum**				
Yes	78 (82.1%)	289 (60.2%)	3.03 (1.74, 5.29)*	2.94 (1.61, 5.37)**
No	17 (17.9%)	191 (39.8%)	1.00	1.00
**Birth at HF before last delivery**				
Yes	24	40	3.72 (2.114, 6.54)*	3.9 (2.04, 7.46)**
No	71	440	1.00	1.00
**Birth order**				
First	23	72	1.00	1.00
Second	19	79	0.75 (0.38, 1.5)	0.12 (0.004, 4.37)
Third	19	95	0.63 (0.32, 1.24)	0.1 (0.002, 3.9)
Fourth and above	34	234	0.46 (0.25, 0.82)*	0.1 (0.003, 3.59)
**History of still birth**				
Yes	4	5	4.18 (1.1, 15.85)*	4.95 (0.73, 33.45)
No	91	475	1.00	1.00

*Statistically significant at p < 0.05 in the crude analysis.

**Statistically significant at p < 0.05 after adjusting for selected confounding variables.

When the obstetric factors; gravida, parity, ANC visit, knowledge of the danger signs of obstetric complications, presence of history of still birth, history of delivery at health facility before last delivery and birth order with socio demographic variables; women’s education and monthly income were adjusted, only ANC visit and history of delivery at health facility before last delivery have significantly associated with birth preparedness and complication readiness (Table 3).

Women who had ANC visits were six times (AOR = 5.68 95% CI = 1.27, 25.41) more likely to prepare for birth and its complication when compared to those who did not have ANC visit. Women who had history of delivery at health facility before last delivery were four times (AOR = 3.9 95% CI = 2.04, 7.46) more likely to prepare for birth and its complication when compared to those who did not have history of delivery at health facility before last delivery (Table 3).

## Discussion

This community based study has attempted to identify the extent and factors associated with birth preparedness and complication readiness in Robe Woreda. The study revealed that proportion of birth preparedness and complication readiness was 16.5% which indicated that birth preparedness was less prevalent in the study area.

The study mainly tried to identify about arrangements made during pregnancy by the mothers for birth and its complication and the result showed that less number of respondents had made arrangement in a comprehensive way prior to the last childbirth commonly by identified a means of transportation, identified skilled provider, saving money and identified place of delivery.

The finding of this study is consistent with previous study [12] and reinforce efforts to increase BP/CR should focus on availing antenatal care services. This result was less than the finding in Adigrat town (22%), which is found in Northern Ethiopia [10] and study done in Northern Nigeria (27.5%) [11]. This difference on preparation made for birth seen between those study populations might be respondents in the current study are rural community while both studies were urban community, since this is may be due to the difference in awareness and educational level of those communities.

One of the most important functions of antenatal care is to offer women advice and information about birth preparedness, danger signs of obstetric complication and emergency preparedness. Birth preparedness is a fundamental component of antenatal care whose aim is to reduce any unnecessary delays to seek emergency obstetric care hence improve maternal and fetal outcomes [1,2,5,14]. In this study any visit to health facility is significantly associated with birth preparedness and complication readiness as previous study [10,12,15,16]. Systematic review and meta-analysis showed that investing in behavior change and community mobilization interventions was reducing maternal and neonatal risks following the concept of “Birth Preparedness and Complication Readiness”, which comprises elements of antenatal care [13]. Those who visit health facilities were six times more likely to prepare for birth and its complication. This could be explained by the fact that those who visit health facility may have the chance to get information on the importance of institutional delivery and to prepare for birth and its complication which can help them to make informed decision on the delivery plan.

Home delivery is norm in many developing countries where mortality tends to be the highest in this case. It is also indicative whether the mother was prepared for birth or not. In the present study less than ten percent of the mothers had given birth in health facilities. This indicates that still majority of the mothers prefer home delivery. This finding is consistent with studies conducted by Ethiopian DHS 2011 for Oromia Region (8.1%) and in South and North Wollo Zone which showed that 5.5% deliveries are managed by health professionals (Habte D, Sheferaw S, Seme A: *Baseline Assessment on Reproductive Health Situation in North and South Wollo Zones of Amhara National Regional State,* Ethiopia; 2007, unpublished). Other community-based survey done 2004 in North Gondor 13.5% of the respondent had delivered in health institution [17]. All of those studies reported that large numbers of women are delivering at home by untrained traditional birth attendants and relatives.

The reasons for not utilizing health service facility for delivery include: short and smooth labor, normal previous home delivery, too much cost of health facility, presence of TBAs, poor quality service of health facility, informed that their pregnancy was normal and health facility too far.

The reasons for not utilizing the service are almost similar with study done 2004 in north Gondor almost half of the respondents reported that labour was short and smooth, needed relative’s attention during labor, facility too far, the presence of TBA and lack of money [17]. Study done in northern Ethiopia, Tanzania, Ghana and Indonesia indicate that some socio-demographic variables and financial limitations were determined whether the community members accessing and using trained attendants and institutional deliveries or not (G/Hiwot F: *Assessment of Factors for Safe Delivery Service Utilization Among Women of Childbearing Age in Ephratanagidim District, North Shoa Zone, Amhara Regional State, Ethiopia*; 2009, unpublished), [18-21].

An important aspect of assessing birth preparedness and its complication readiness is measuring spontaneous knowledge of essential danger signs of obstetric and newborn complications. Knowledge of the danger signs of obstetric complications is the first step in the appropriate and timely referral for essential obstetric care [2,5]. The spontaneous knowledge of respondents about key danger signs is higher when compared with other study [10,16]. This indicates that good awareness of women and a possible high chance of good outcome of pregnancy. This could be attributed by HEWs to be aware about obstetric complications and to promote utilization of health care services. However, mothers may not practice all what they know due to different factors.

Knowledge of danger signs of obstetric complications are an essential step in recognition of complications and enable one to take appropriate action to access emergency care. Approximately 25% of maternal deaths occur due to severe vaginal bleeding as obstetric complication worldwide [2,5,6].

Vaginal bleeding which is a danger sign of obstetric complication was not known by almost half of the respondents. This is worrying given that hemorrhage is the leading cause of maternal mortality worldwide and responsible for 33% of all maternal deaths [5]. Equally worrying was the inability of the most respondents to identify danger signs which indicate severe pre-eclampsia and eclampsia such as blurred vision and swelling of hands/face.

About 45.6% of the respondents reported that they identified place of delivery ahead of childbirth. Place of delivery identification is very important especially in this setting where the main means to get a skilled provider is to deliver at health institutions. But, among mother’s who identified place of delivery (45.6%), majority of the mothers (40.7%) had planned to give birth at home while only (4.9%) of the mothers had planned to give birth at the nearest health facility. Still this indicated that majority of the mothers want to give birth at their home which was similar with other study [16]. The reasons for these may be as found in qualitative study were the trust and tradition on TBA and the services of trained birth attendants during childbirth or an institutional delivery were perceived important by some community members only during obstetric complications.

Lack of money and transportation is a barrier for seeking care as well as identifying and reaching medical facilities [5,6]. Money saved by woman or her family can pay for health services and supplies, vital for transport, or other costs. Likewise, if a woman can afford to pay for these costs, she is more likely to seek care [5]. In the present study, three-forth of the respondents saved money for childbirth which is higher compared to a study in Adigrat (68.9%) [10]. This could be due to the cultural value of the community in the study area that is report as they prepare or save money even if they do not save; means social desirability bias. Even when money is available, it can be difficult to secure transport at the last minute after a complication has occurred.

Arranging transport ahead of time reduces the delay in seeking and reaching services. In this study, almost one-third of the respondents has identified transportation ahead of childbirth which is higher compared to a study in Adigrat (24.7%) [10]. This could be due to difference in transport type and increased awareness of mothers by HEWs towards identifying transportation ahead of childbirth to health facilities and may also as there is longer period between the two studies. But, our study was less when compared with study done in West Bengal, India [22]. This difference may be due to difference in awareness level and transportation type.

Educated mothers were six time more likely to be prepared for birth and its complication than illiterate. As study in India, Ethiopia and Nigeria showed, mother’s education had large positive effects on the institutional delivery which was the outcome of BP/CR [20,23-25]. According to community based study done in Ethiopia, Gulele district, Addis Ababa, also indicate that the risk of choosing to deliver at home was found high for those who are illiterate [26]. Similarly, the study done in Arsi zone central Ethiopia indicate, women who had no formal schooling are found to attend antenatal care less likely [27]. It is obvious that more educated mothers tend to have better awareness on warning signs of obstetric complications. It also might be related to the fact that educated women have better power to make their own decision in matters related to their health and the expected expenses.

Consistent with different studies [11,28,29] monthly income also found to be predictor for birth preparedness and complication readiness. Mothers who had better income were two times more likely to prepare for birth and its complication than mothers who had less monthly income. These could be economic status of mother is able to make wise decision and payment by her own than their counterparts.

Having knowledge of obstetric complications was found to have statistical significance with birth preparedness and complication readiness both in this study and other similar studies done in Ethiopia and other places [10,11,23,28,29]. The reason for this might be mothers with knowledge of obstetric complications may fear something may happen and need advice and support from health personnel.

Women who had ever given birth in the health facility before were four times more likely to prepare for birth and its complication. This was consistent with other studies [11,28,29]. This strong significant association could partly be explained due to increased in mothers’ confidence and trust on providers from previous use of the services.

The strengths of this study is that purely rural community based which was supplemented by the qualitative study and data collectors were extensively trained similar sex. On the other hand, since the study was cross sectional, temporal relationship could not be established. The findings are self-reported; therefore, there can be some recognition and recall bias. To minimize recall bias, we selected mothers with infants of age 12 months and lower.

Further research should be conducted on quality of maternal health services; particularly the reason for gaps between ANC, BP/CR and delivery service utilization as well as provider’s knowledge, practice and attitude towards BP/CR.

## Conclusions

Based on the findings of the research it is concluded that the magnitude of BP/CR in the study area is very low (16.5%). The principal factors affecting BP/CR were women’s educational status, ANC visits and women who have knowledge of obstetric complications. Other factors which were significantly associated with BP/CR were women those who have given birth at health facility before their last delivery and those who have better monthly income. The study has also clearly evidenced that the respondents’ knowledge of danger signs was low and large proportion of clients were not prepared for obstetric emergencies.

Thus, community-based education about preparation for birth and its complication and empowerment of women by expanding educational opportunities are important factors in enhancing birth preparedness and hence reducing the effect of pregnancy related complications.

Antenatal care clinics should give due emphasis to preparation for birth and its complication and provide information and education to all pregnant women. Birth preparedness and complication readiness should be made an integral part of maternal and child health services, to enable women to recognize danger signs and access to skilled caregiver during pregnancy and child birth.

## Competing interests

We declare that there are no financial or non-financial competing interests related to this study.

## Authors’ contributions

MK was taken a lead role in writing the proposal, submission and follow up for ethical review, data collection, data entry, and writing of the preliminary results. MK was also responsible in writing up and for the final approval of the manuscript to be published. MA was participated in the planning of the study by giving constructive comments and idea. MA has been involved significantly in the analysis and writing of the manuscript through commenting. MA was responsible for writing up and final approval of the manuscript to be published. Both authors read and approved the final manuscript.
